# Corneal epithelial cells exposed to shear stress show altered cytoskeleton and migratory behaviour

**DOI:** 10.1371/journal.pone.0178981

**Published:** 2017-06-29

**Authors:** Sara Molladavoodi, Matthew Robichaud, David Wulff, Maud Gorbet

**Affiliations:** 1 Department of System Design Engineering, University of Waterloo, Waterloo, Ontario, Canada; 2 Department of Chemical Engineering, University of Waterloo, Waterloo, Ontario, Canada; Universite de Technologie de Compiegne, FRANCE

## Abstract

Cells that form the corneal epithelium, the outermost layer of the cornea, are exposed to shear stress through blinking during waking hours. In this *in vitro* study, the effect of fluid shear stress on human corneal epithelial cells (HCECs) was investigated. Following exposure to shear stresses of 4 and 8 dyn/cm^2^, HCECs showed cytoskeletal rearrangement with more prominent, organized and elongated filamentous actin. Cytoskeletal changes were time-dependent, and were most significant after 24 hours of shear stress. Higher rates of migration and proliferation, as evaluated by a scratch assay, were also observed following 24 hours of low shear stress exposure (4 dyn/cm^2^). This result contrasted the poor migration observed in samples scratched before shear exposure, indicating that shear-induced cytoskeletal changes played a key role in improved wound healing and must therefore precede any damage to the cell layer. HCEC cytoskeletal changes were accompanied by an upregulation in integrin β_1_ and downregulation of ICAM-1. These results demonstrate that HCECs respond favourably to flow-induced shear stress, impacting their proliferation and migration properties as well as phenotype.

## Introduction

It is well recognized that mechanical signals can affect cell behaviour including proliferation, migration, and differentiation [1–3]. The process by which cells detect and respond to mechanical signals is referred to as mechanotransduction [4]. Various mechanical stimuli have been previously shown to affect cell behavior, including external forces such as tensile and compressive stresses [5] as well as mechanical properties of the substrate itself (such as stiffness) [6]. Cells are also known to respond to shear stress as a mechanical signal [7–10]. Its effects on endothelial cells [7,11,12] and leukocytes [13–15] have been well-documented.

The cornea is the most important refractive component of the ocular system and is often referred to as the window of the eye, with the corneal epithelium being its outermost layer [16]. The cornea is exposed to various mechanical stimuli; matrix stiffness [17] and topography [18,19] have been shown to affect keratocytes and corneal endothelial cells. Furthermore, we previously observed that increased substrate stiffness led to changes in cytoskeletal structure and increased migration speed in corneal epithelial cells [20]. Leonard *et al*. [21] found that applying axial strains on rabbit corneal fibroblasts can affect α-smooth muscle actin (α-SMA) expression. In their study, strain in the physiological range (less than 7%) downregulated α-SMA expression, which was believed to contribute to corneal transparency [21]. It has also been suggested that shear forces due to blinking can affect corneal epithelial cell size and migration patterns [22]. However, while corneal epithelial cells are known to be exposed to shear stresses caused by eyelids during blinking and eye rubbing [23], little is known about its effects on cell phenotype. Using an “*in vitro* whole-eye perfusion” model, applying shear stress to the surface of rabbit corneas led to changes in corneal epithelial cell morphology and increased shedding rate [24]. Additionally, using a cone and plate model, rabbit corneal epithelial cells exposed to shear stress were found to have increased ATP release [25].

In the limited number of studies on corneal epithelial cells, shown in Table 1, experimental conditions differ greatly. The magnitude of flow-induced shear stress experienced by corneal epithelial cells due to blinking remains an active area of investigation, with significant variation in proposed values ranging from 0.05 [26] to 14 dyn/cm^2^ [25]. To support the development of better therapeutic strategies to preserve vision, further *in vitro* studies are required to gain a better understanding of how HCECs may respond to the flow-induced shear stress induced by blinking. Previous *in vitro* studies with HCECs [24–26] and other cells [7,11–15] suggest that shear stress may affect cell morphology and growth, we therefore hypothesized that exposing human corneal epithelial cells to shear stress may result in cytoskeletal migratory changes. In this study, the response of human corneal epithelial cells (HCECs) to two levels of shear stress, 4 (low) and 8 dyn/cm^2^ (high), was investigated. Expression of membrane receptors and apoptosis markers were evaluated, as well as cytoskeletal and migratory changes using a scratch assay.

**Table 1:** Experimental conditions of *in vitro* studies of corneal epithelial cells exposed to flow-induced shear stress.

In vitro study	Cell type	Shear stress magnitude and *in vitro* flow model	Exposure time
Srinivas *et al*. [25]	Primary rabbit corneal epithelial cells	0.34 dyn/cm^2^ rotating disk model	20 sec
Kang *et al*. [26]	Limbal epithelial stem cells (LESCs)	0.07 dyn/cm^2^ parallel plate flow channel	2 hours intermittent flow/day for 2 days
Pretor *et al*. [27]	SV-40 immortalized corneal epithelial cells	1 dyn/cm^2^ microfluidic flow channel	40 min
Ren & Wilson [24]	Whole rabbit eyes	Magnitude not reported, magnetic stirring of solution on inverted corneas.	6 hours

## Materials and methods

### Cell culture

HPV-immortalized human corneal epithelial cells (HCECs), previously gifted by May Griffith, were used in this study [28]. Cells were maintained in keratinocyte medium (KM; ScienCell, Carlsbad, CA, USA), supplemented with keratinocyte growth supplement (KGS; ScienCell, Carlsbad, CA, USA) and Penicillin/Streptomycin (ScienCell, Carlsbad, CA, USA) at 37°C, 5% CO_2_, and 95% humidity. The cell culture medium was replaced every 2–3 days. Only cells below passage eleven were used.

Primary human corneal epithelial cells were also used in some experiments (cytoskeleton study) and were purchased from ScienCell (Carlsbad, CA, USA). Primary HCECs were treated similarly to immortalized HCECs, although only the first three passages were used.

### Sample preparation

Glass coverslips (#2, 35mm diameter; Glycotech, Gaithersburg, MD, USA) were coated with 0.05 mg/ml rat tail collagen type I (ScienCell, Carlsbad, CA, USA) at room temperature under aseptic conditions. Following incubation for 45 minutes, coverslips were washed three times with PBS, and 5×10^5^ cells were seeded on the coverslips. To allow for cell attachment on the coverslips, samples were incubated for 30 minutes at 37°C, 5% CO_2_, and 95% humidity. Remaining medium was then added and coverslips were incubated for 18–24 hours prior to the start of the experiment to ensure confluence of the monolayer.

### Experimental setup

**Fig 1:**
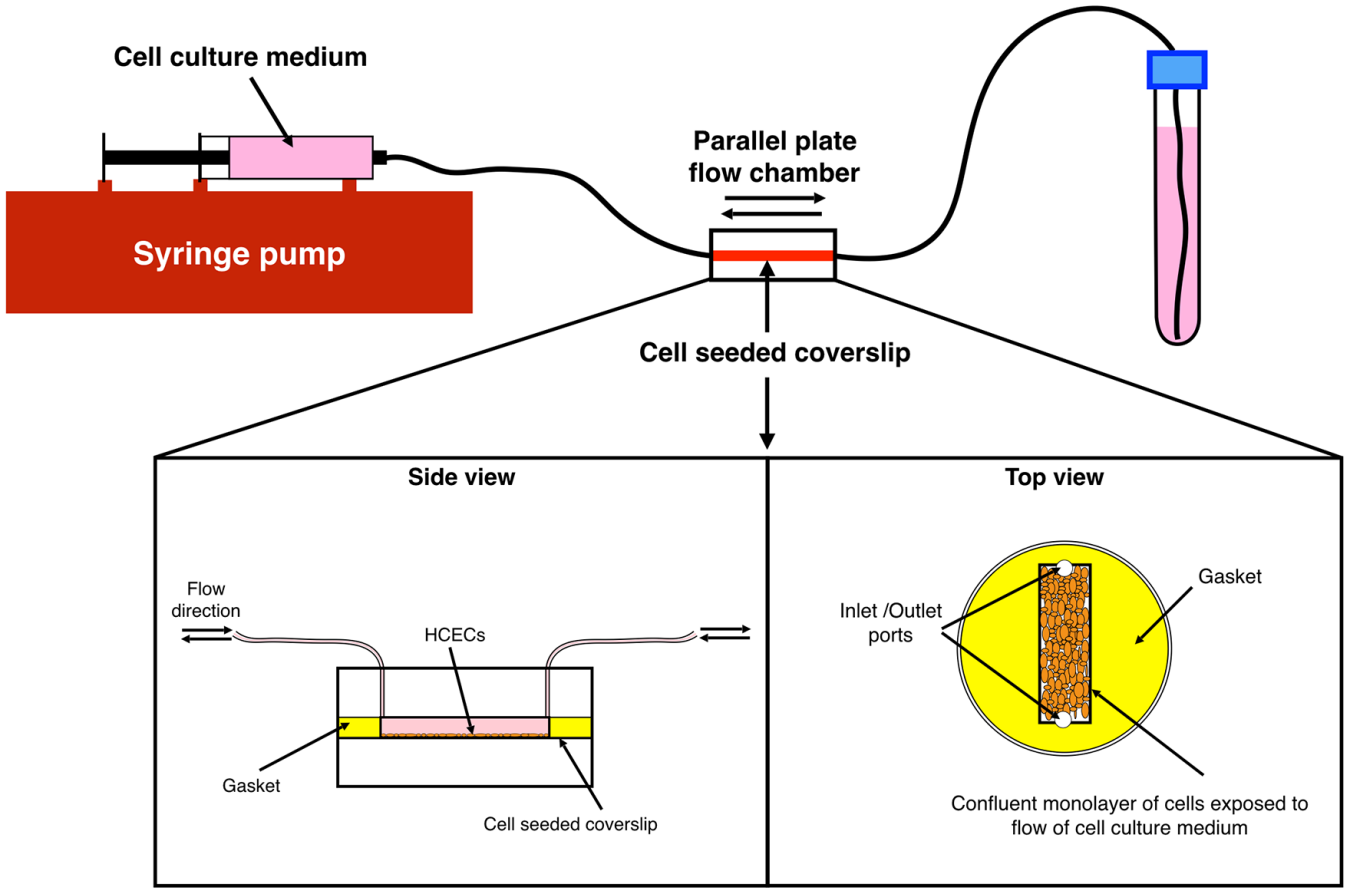
Schematic of the experimental setup for applying shear stress to HCECs using flow of cell culture medium. Shear stresses of 4 and 8 dyn/cm^2^ were applied to cells for 6, 14, and 24 hours. Schematic of side and top views of the parallel plate flow chamber, as well as the cell-seeded coverslips and gasket in the chamber can also be seen.

Flow-induced shear stress was applied to the cells using a controlled flow rate of cell culture medium over the cell monolayer. The cell-seeded coverslips were placed in a parallel plate flow chamber (Glycotech, Gaithersburg, MD, USA). Bi-directional flow of cell culture medium (KM) was applied to the cells using a syringe pump (Cole-Parmer, Montreal, QC, Canada). Fig 1 presents a schematic of the experimental setup using the parallel plate chamber.

The flow rate was set at 3.6 ml/min and gaskets with different widths were used to create two levels of shear stress, 4 and 8 dyn/cm^2^, as calculated using the Newtonian laminar flow equation (Eq 1) and the dimensions of the gaskets (length: 20 mm, width: 5 and 10 mm, and height: 0.254 mm).

$$\tau_{mean}=\;\frac{6\mu Q}{bh^{2}}$$ (1)

where μ is the dynamic viscosity of the culture medium (dynamic viscosity of water at 37°C was used, 6.92 × 10^−4^ kg/(m·s)), *Q* is the flow rate, and *b* and *h* are width and height of the gaskets, respectively.

**Fig 2:**
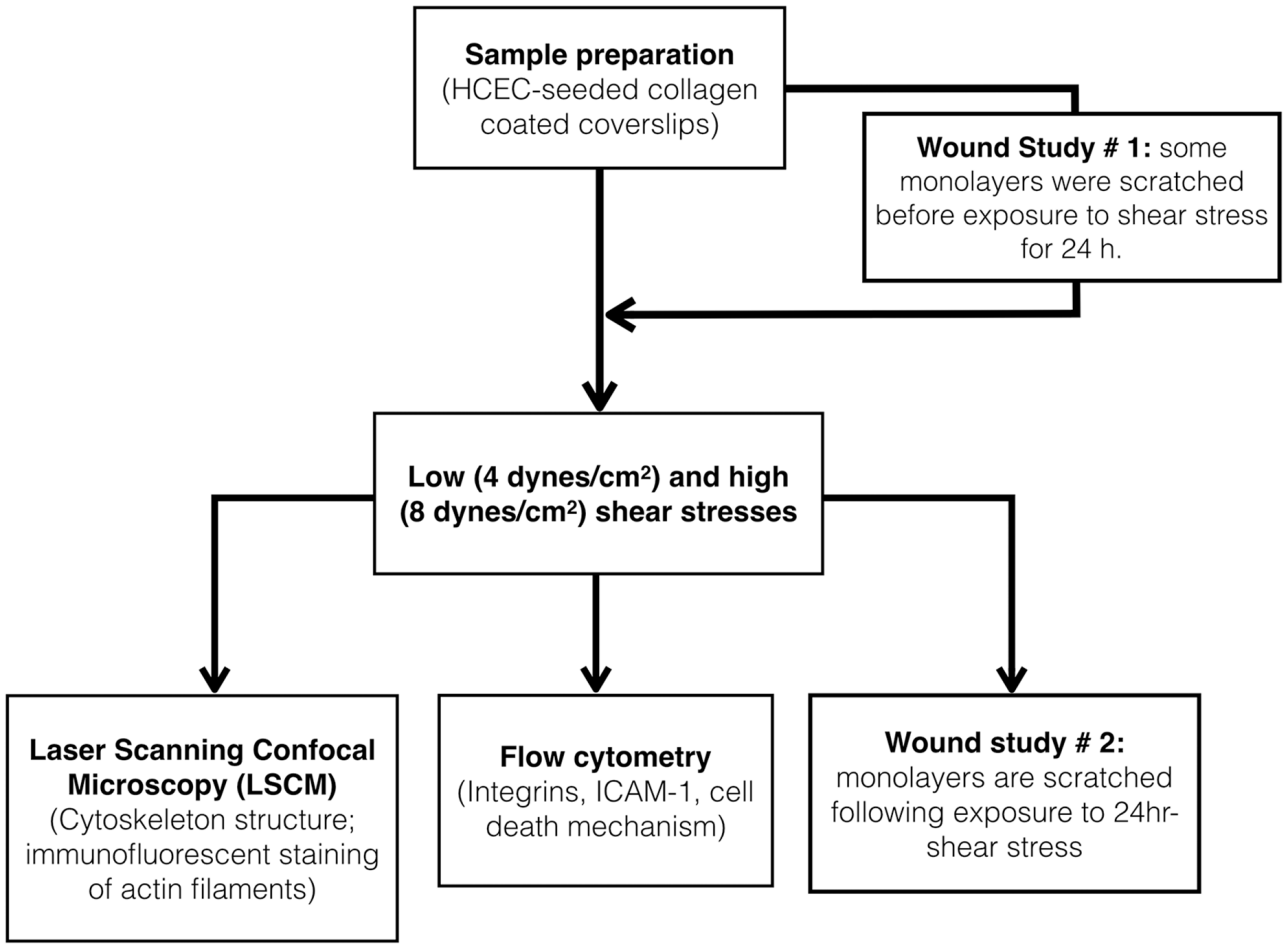
Diagram showing experiments and the sequence in which they are performed in this study.

Cells were exposed to these two levels of flow-induced shear stress for 6, 14, and 24 hours. Cells seeded on collagen-coated coverslips but not exposed to shear stress were used as controls. Fig 2 presents a diagram of the sequence of experiments performed in the study.

To visualize flow in the chamber, water mixed with red food coloring was passed through the system under similar experimental conditions. The flow of the liquid in the chamber for 4 dyn/cm^2^ during both pumping and retracting (changing flow direction) and the absence of significant turbulence can be seen in S1 Movie.

### Flow cytometry

Flow cytometry was used to investigate changes in expression of integrin-α_3β1_ and intercellular adhesion molecule-1 (ICAM-1) as well as apoptosis. Following the experiments, cells were detached from the coverslips using TripLE^™^Express (Thermo Fisher Scientific, Waltham, MA, USA). To ensure that only cells exposed to shear stress were collected, cells were detached before flow chamber disassembly. After gentle washing following TripLE^™^Express treatment, cells were incubated with fluorescently labelled antibodies against integrin β_1_ (CD29), integrin-α_3_ (CD49c) and ICAM-1 (CD54) (BD Biosciences, USA) for 30 minutes at room temperature in the dark. Samples were then diluted and fixed using paraformaldehyde (1% final concentration) and analyzed by flow cytometry (BD FACSCalibur, BD Biosciences, San Jose, CA, USA) within 5 days.

Caspase-mediated apoptosis and necrosis were assessed using FAM-FLICA^™^ *in vitro* poly caspases kit (Immunochemistry Technologies, Bloomington, MN, USA). Following the manufacturer’s protocol, cells were incubated with the polycaspase enzyme probe, FAM-VAD-FMK. After 1 hour of incubation at 37°C, cells were washed three times with PBS. Propidium iodide (PI) (Immunochemistry Technologies, Bloomington, MN, USA) was added immediately prior to flow cytometric analysis.

### Laser scanning confocal microscopy

To investigate the effects of flow-induced shear stress on the cytoskeleton structure of HCECs, cells were first fixed using 4% paraformaldehyde and permeabilized with 0.1% Triton solution. Nonspecific binding was blocked with a 1% BSA solution prior to staining. Actin filaments were stained using Alexa Fluor^®^ 488 Phalloidin (Molecular Probes, Thermofisher Scientific, Waltham, MA, USA).

Expression and localization of integrin β_1_ was also investigated. Control cells and cells exposed to low shear for 24-hr were fixed with 4% paraformaldehyde. Samples were permeabilized with 0.1% Triton and nonspecific binding was blocked with 1% BSA/10% goat serum. Samples were incubated with the monoclonal antibody against β_1_ (ab24693, Abcam) followed by Alexa Fluor^®^ 488 conjugated secondary antibody (ab150113, Abcam).

Images were taken with an inverted laser scanning confocal microscope (LSCM; Carl Zeiss, Oberkochen, Germany), using an argon laser at a wavelength of 488 nm.

### Scratch wound experimental model

The effects of flow-induced shear stress on HCEC migration and proliferation was studied using a scratch assay. To better understand the effects of shear stress, two experimental studies were performed (see Fig 2):

1. In experimental study #1, the monolayer was first scratched and then immediately exposed to shear stress for 24 hours. The cells were then incubated under static conditions at 37°C, 5% CO_2_, and 95% humidity for up to 3 days until wound closure.
2. In experimental study #2, the monolayer was first exposed to shear stress for 24 hours and then scratched. The cells were then incubated under static conditions at 37°C, 5% CO_2_, and 95% humidity for up to 3 days until wound closure.

**Fig 3:**
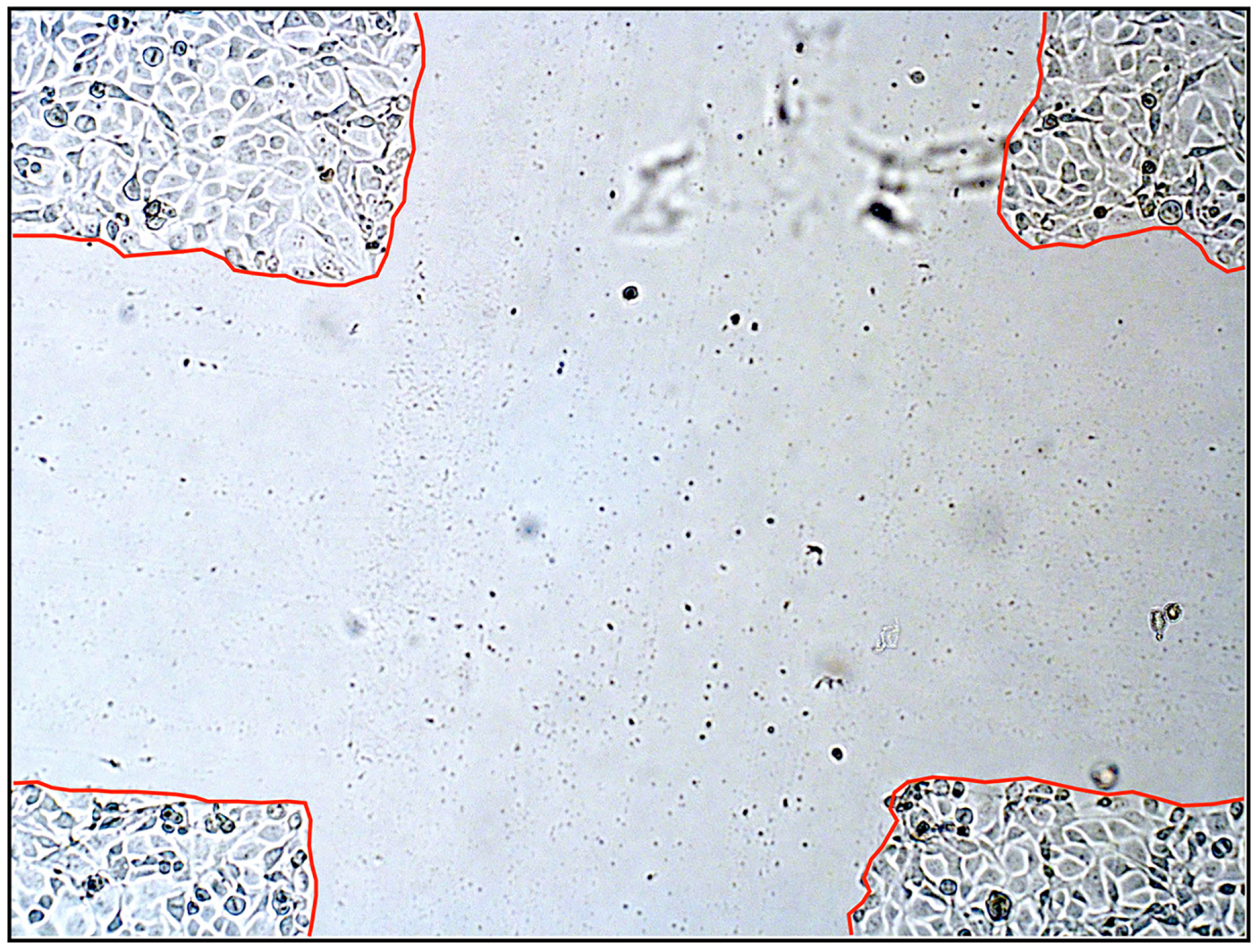
Optical micrograph of the scratch pattern used in this study.

To ensure micrograph measurements were performed consistently across the wounded area and easier localization of the wounded area, a plus sign was used as the scratch pattern for this study (Fig 3) (Wattsky and Lu, *Invest*. *Ophthalmol*. *Vis*. *Sci*. *55* (2015), E-abstract 5625). Pictures were taken daily using a Nikon Eclipse inverted microscope (Nikon, Melville, NY, USA). Width measurements were done on micrographs using the NIS-Elements software (Nikon) by averaging the widths of the scratched channels in each arm of the plus sign. At day 0, the day the scratch was created, measurements were taken and all following measurements (24, 48, 72 hours) are presented as a percent of the original (day 0) average scratch width.

To confirm the role of β_1_ integrin in wound healing, experiments were performed where an anti-β_1_ integrin blocking antibody was added to the medium (final concentration of 5μg/ml) following initial shear exposure (4 dyn/cm^2^, 24 hrs) and wounding, and healing was observed for up to 48 hours. Controls included shear and no shear samples without the blocking antibody.

### Statistical Analysis

Statistical analysis was performed using RStudio (RStudio, Inc. Boston, MA, USA). Analysis of variance (followed by a multiple pairwise comparisons using Tukey’s HSD test) was used to calculate statistical significance. The significance level was considered to be 0.05.

## Results and discussion

### HCECs exposed to flow-induced shear stress have reorganized cytoskeletons

**Fig 4:**
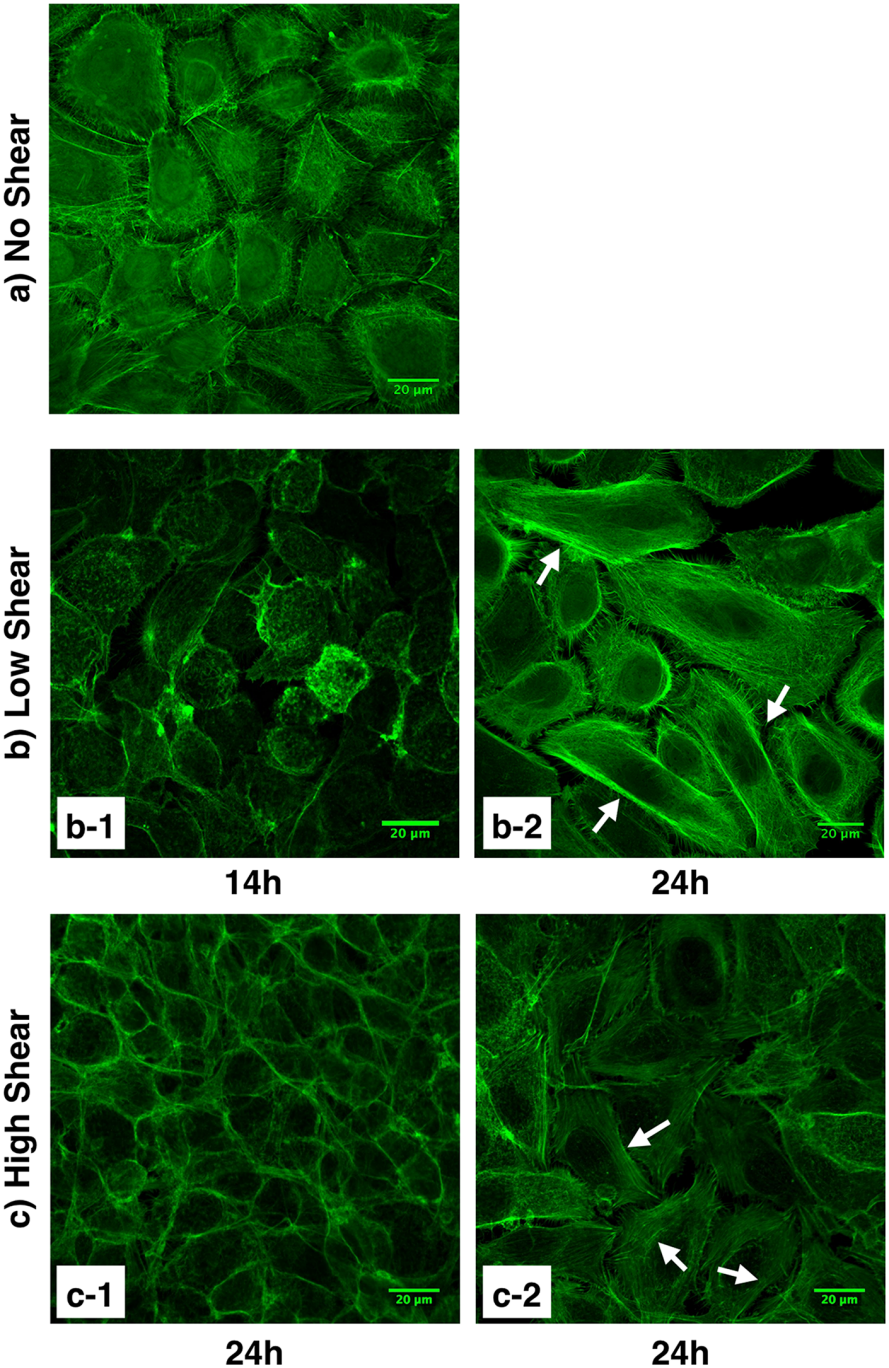
HCEC cytoskeleton organization. Actin filaments were stained with fluorescently labeled phalloidin. a) Control cells (i.e., not exposed to shear stress). b) Cells exposed to low shear stress (4 dyn/cm^2^) for 14 hours (b-1) and 24 hours (b-2). Organization of the cytoskeleton with actin filament bundles are visible in cells exposed to low levels of shear stress for 24 hours. c) Cells exposed to high shear stress (8 dyn/cm^2^) for 24 hours with less visible (c-1) and more visible (c-2) filamentous actin cytoskeleton structure. White arrows indicate stretched actin filaments. Images captured using a Zeiss laser scanning confocal microscope.

**Fig 5:**
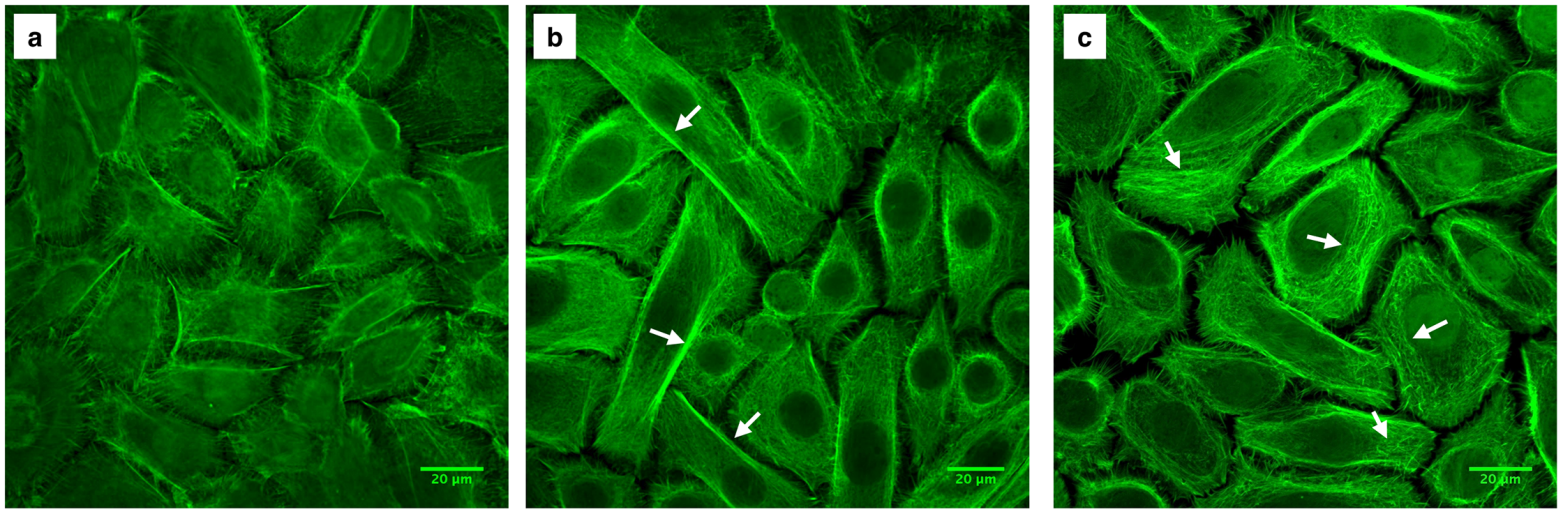
Primary HCEC cytoskeleton organization. Actin filaments were stained with fluorescently labeled phalloidin. a) Control cells (i.e., not exposed to shear stress). b) Cells exposed to low shear stress (4 dyn/cm^2^) for 24 hours; white arrows indicate the stretched organized actin filaments, c) Cells exposed to high shear stress (8 dyn/cm^2^); white arrows indicate actin filament formation. Images captured using a Zeiss laser scanning confocal microscope.

The cell cytoskeleton not only allows cells to mechanically interact with their environment, it is also involved in maintaining cell shape, growth, division, and migration. As one of the three types of filaments forming the cell cytoskeleton, actin filaments have been previously shown to be involved in mechanotransduction pathways as mechanosensors [29]. Actin filaments were stained with fluorescently labeled phalloidin, and as shown in Fig 4, cytoskeletal reorganization occurred in HCECs exposed to shear stress. These changes were dependent on exposure time and visible cytoskeleton reorganization was observed after exposure to flow-induced shear stress for 24 hours. In the control HCECs (i.e. not exposed to shear stress) (Fig 4a), short, disorganized actin filaments were mainly gathered around the cell perimeter. Although some longer actin filaments were observed, similar organization of the filaments around the membrane occurred in cells exposed to shear stress for 14 hours (Fig 4b-1). After 24 hours of shear exposure, cells displayed stretched and organized actin filament bundles inside the cells (white arrows, Fig 4b-2). It is worth noting that although changes in cytoskeleton organization were seen in samples exposed to high (8 dyn/cm^2^) and low (4 dyn/cm^2^) shear stresses, reorganization was most prominent and uniform for cells exposed to the low shear condition. For cells exposed to higher shear, cytoskeleton reorganization was less consistent, and both areas of cells with less visible actin filaments (Fig 4c-1) and areas of cells with distinguishable more organized actin filaments (Fig 4c-2) were observed. Cytoskeleton rearrangement in response to flow-induced shear stress was further confirmed with primary corneal epithelial cells (Fig 5). Primary HCECs exposed to low levels of shear stress for 24 hours displayed stretched actin filaments (Fig 5b) while cells exposed to high levels of shear stress formed smaller actin filaments that gathered around the perimeter of the cell and lacked organized areas of actin bundles (Fig 5c).

Cytoskeletal reorganization following flow-induced shear stress has been previously reported for endothelial cells [11,12,30]. Galbraith et al. [11] have reported the time-dependency of cytoskeletal reorganization for endothelial cells as well. In endothelial cells, cytoskeletal rearrangement has been related to different intracellular signaling pathways [12,31,32], which recruit many molecules including focal adhesion kinases (FAK) and mitogen-activated protein (MAP) kinases [33]. Differences in cytoskeletal organization have also been reported to depend on the level of shear stress and direction [34–36].

While many studies have assessed the effects of physical cues on epithelial cells such as surface topography [37–40] or external electric fields [41,42], to the best of our knowledge, this is the first study to report these cytoskeletal changes in corneal epithelial cells due to flow-induced shear stress.

### HCECs with reorganized cytoskeletons heal wounds more quickly

**Fig 6:**
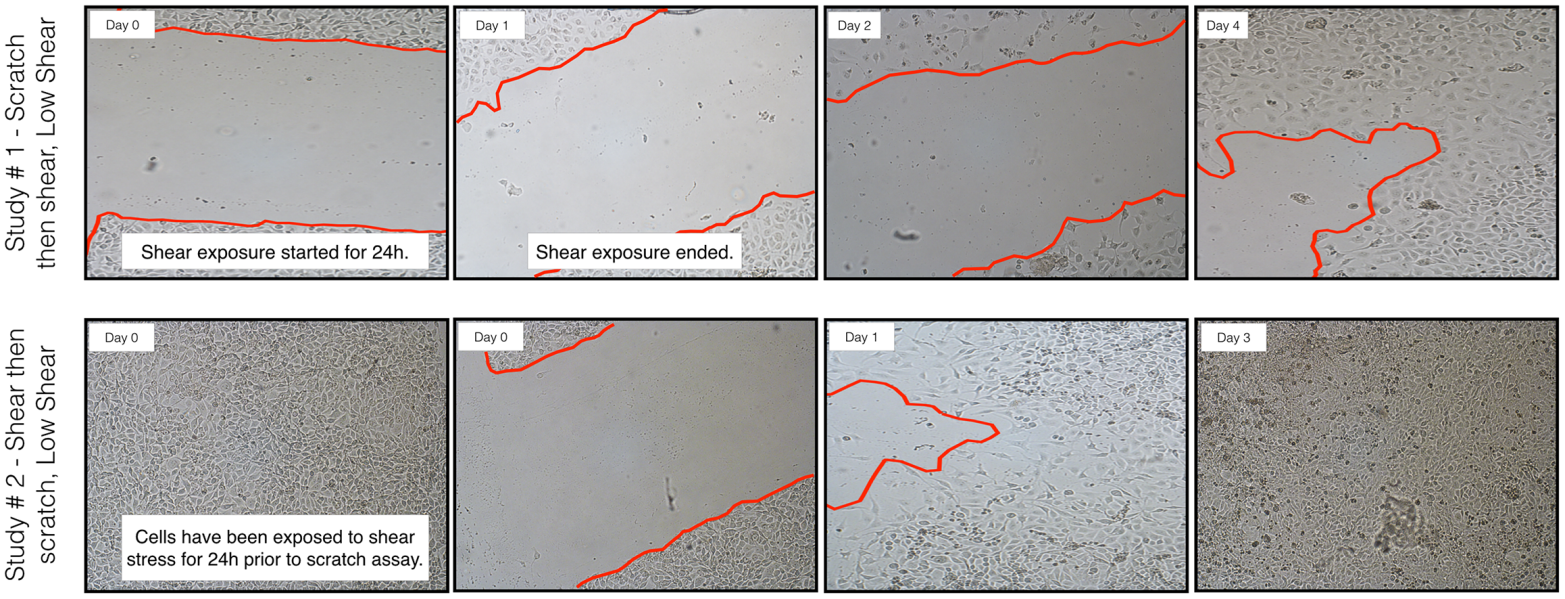
Effect of shear stress on migration of HCECs in a scratch wound *in vitro* model. Optical micrographs of changes of wound width over time. a) Study #1, confluent monolayers were scratched and then immediately exposed to 4 (low) or 8 (high) dyn/cm^2^ for 24 hours. b) Study #2, confluent monolayers were exposed to 4 (low) or 8 (high) dyn/cm^2^ for 24 hours and then scratched. Wound width was assessed for up to 3 days after wounding. Optical micrographs were taken using a Nikon inverted optical microscope.

**Fig 7:**
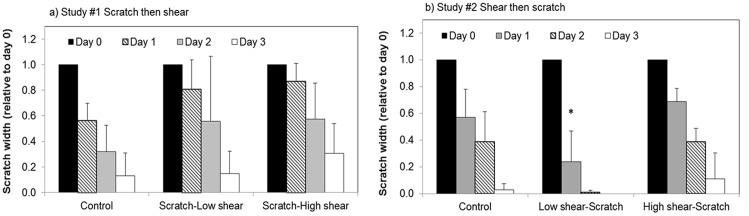
Effect of shear stress on wound healing. Changes in wound width were measured daily and are reported normalized to scratch width on Day 0. a) Scratch then high/low shear: confluent monolayers were scratched and then immediately exposed to 0 (control), 4 (low) or 8 (high) dyn/cm^2^ for 24 hours and wound width was then assessed. b) High/low shear then scratch: confluent monolayers were exposed to 0 (control), 4 (low) or 8 (high) dyn/cm^2^ for 24 hours and then scratched. N = 3 to 5 (except for day 3 shear, n = 2 to 4), mean ± standard deviation. * significantly different from control (no shear), *p* < 0.05.

As significant cytoskeletal changes were observed after 24 hours of exposure to shear stress, this exposure time was selected to further investigate the effect of shear stress on cell migration in wound healing using a scratch assay. As observed in Fig 6a, in the shear stress-exposed “wounded” sample (Study #1—scratch then shear), HCECs were not able to effectively proliferate and migrate to heal the wound. When shear was performed after scratching, wound healing was impaired as evidenced by a lack of significant reduction in scratch width in both low and high shear samples up to day 2 after wounding/shear exposure, compared to the no-shear control (Fig 6a). Conversely, HCECs exposed to shear stress prior to scratching (Study #2, shear then scratch, Figs 6b and 7b), showed significantly increased migration and proliferation. This was particularly true for cells exposed to low shear (as shown in Fig 4b-2), which healed the wound to nearly 75% within 24 hours (Fig 7b).

The results of study #1, wherein shear stress was observed to be detrimental to wound healing when a wound was created in a confluent monolayer (not previously exposed to shear) concur with the results of a newly published study by Utsunomiya *et al* [43]. It is however important to highlight that, as HCECs are regularly exposed to shear stress through blinking, exposing cells to shear stress for the first time following wounding may not fully capture the role of shear stress in corneal epithelial cells. The significant difference in healing between study #1 and study #2, where shear stress was applied either before (study #2) or after (study #1) the wound, points to the critical role of cytoskeleton organization in wound healing. The organization of the cytoskeleton components such as actin filaments is a key player in proliferation and migration [29,44]. This has been shown previously for corneal epithelial cells [20,45,46]. Using live cell imaging, we have previously demonstrated that HCECs without stretched and visible actin filaments were not capable of migrating effectively [20]. As opposed to the increased healing observed following exposure to low shear stress, exposing cells to high shear stress prior to wounding did not lead to significant changes in scratch width reduction, which could be explained by the non-uniform cytoskeletal rearrangement observed following high shear stress exposure. The effects of cytoskeleton organization on the migration and proliferation of epithelial cells have been previously reported by Yin et al. [47,48]. Cytoskeletal reorganization has been linked to small GTPase Rho and its downstream effector Rho-associated protein kinase (ROCK) [48].

Additional experiments were performed to determine whether wound healing would be impaired if study #2 (shear-then-scratch) was followed with additional shear exposure. Six hours of low shear stress (4 dyn/cm^2^) after wounding was chosen for the additional exposure time; it was desirable to minimize additional stress on the cells given the already significant manipulative stress (growing the monolayer on a coverslip, mounting in the parallel flow chamber, 24 hours shear exposure, dismantling the flow chamber, scratching the monolayer and repeating flow chamber assembly). Following additional shear stress exposure, the cells showed faster wound closure compared to samples without any shear stress (34% versus 21% after one day, n = 3) and by day 2, all shear samples exposed to additional shear after wounding were fully healed. These results provide further indication that shear stress does not unconditionally impair wound healing as suggested by the results of Utsunomiya *et al* [43]. To determine the role of shear stress in wound healing, the effect of physiological shear stress on cell phenotype, such as cytoskeletal reorganization, must first be taken into consideration; exposing cells to shear following damage does not mimic the physiological environment where corneal epithelial cells have been exposed to blinking consistently and thus have adopted a phenotype associated with exposure to shear stress. It is also important to note that the current *in vitro* models used to assess corneal epithelial cell response to shear stress in previous work [25–27,43] and even our own are limited as they have not been designed to mimic the physiological ocular environment. New *in vitro* models will need to be developed to better characterize the corneal epithelial cell response to shear stress.

### Flow-induced shear stress differentially affects cell-substrate adhesion molecule expression in HCECs

Integrins are heterodimer molecules consisting of α and β subunits important in cell-substrate adhesion [49]. In epithelial cells, integrin α_3_ (CD49c) exclusively heterodimerizes with β_1_ (CD29), and integrin α_3_β_1_ is involved in cell migration, especially during wound healing [50,51]. Integrin α_3_β_1_ is also important in adhesion, cell spreading, and cell-substrate interactions [52,53]. Furthermore, this molecule has been associated with epithelial cell proliferation [52]. A flow cytometry study was thus undertaken to determine if shear stress-induced changes in integrin expression accompany the observed cytoskeletal changes.

As shown in Table 2, while integrin α_3_ expression was relatively constant in all conditions, higher expression of integrin β_1_ was observed in cells following exposure to shear stress when compared to control cells, with significant upregulation following exposure to low shear (4 dyn/cm^2^) for 24 hours (*p* = 0.03). The changes in integrin β_1_ expression in HCECs following exposure to shear stress appear to be in concurrence with the cytoskeletal changes described above (Fig 4). Upregulation and relocalization of integrin β_1_ have been previously linked to mechanoresponse [54] and cytoskeleton reorganization [55] in endothelial cells exposed to flow-induced shear stress. Shear stress has also been reported to change the state of integrin bonds to the surface, whereby integrin/ligand bonds can change from “relaxed” to “tensioned” under the external force, which can in turn contribute to the observed cytoskeletal reorganization [56].

**Table 2:** Effect of shear stress and exposure time on the expression of integrin α_3_, β_1_ and ICAM-1 in HCECs.

Marker	Shear stress	6 Hours	14 Hours	24 Hours
Integrin α_3_	Low Shear	1.13 ± 0.12	1.04 ± 0.11	1.22 ± 0.34
	High Shear	1.08 ± 0.16	1.01 ± 0.13	1.04 ± 0.16
Integrin β_1_	Low Shear	1.32 ± 0.55	1.35 ± 0.31	1.54 ± 0.17*
	High Shear	1.38 ± 0.56	1.37 ± 0.42	1.38 ± 0.28
ICAM-1	Low Shear	0.91 ± 0.07	0.91 ± 0.09	0.77 ± 0.09*
	High Shear	0.91 ± 0.09	0.91 ± 0.07	0.81 ± 0.11

HCECs were exposed to low and high shear stress for 6, 14 and 24 hours. Expressions are normalized to the control sample (no shear) and reported as relative value. N = 3 to 4, mean ± SD.

*significantly different from control (no shear), *p* < 0.04.

**Fig 8:**
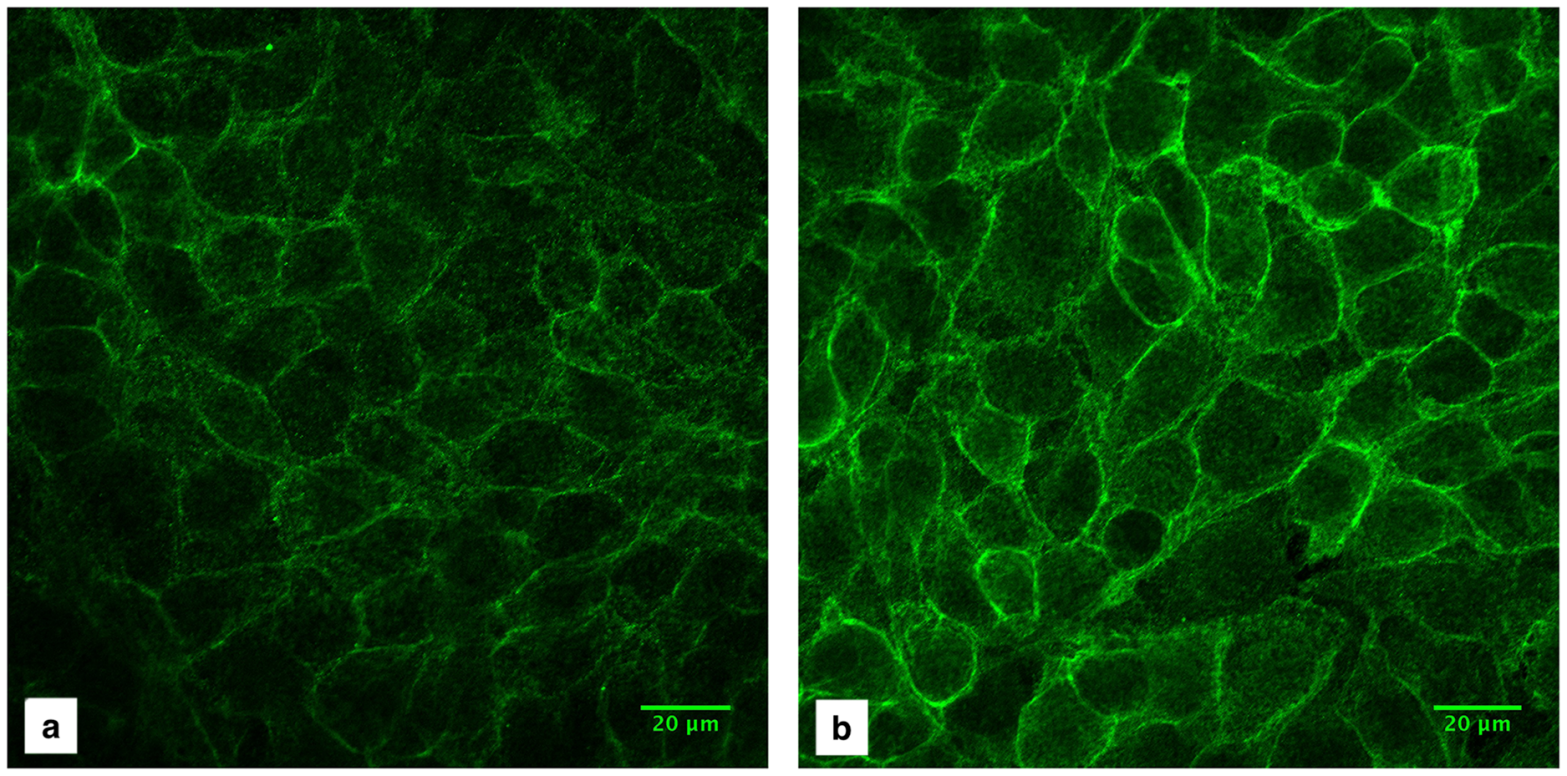
Effect of shear stress on integrin β_1_ expression. a) Control (no shear) sample, b) After 24 hours of low shear stress exposure.

Several experiments were performed to further explore the modulation of integrin β_1_ following shear and its role in wound healing. In order to determine if there were changes in its localization, samples exposed to the low shear or no shear condition were stained with anti-integrin β_1_ and imaged using identical settings to allow direct comparison. As shown in Fig 8, higher intensity for integrin β_1_ staining can be observed in the low shear sample compared to no shear, further confirming our flow cytometry results. The mean pixel intensity of the shear sample (Fig 8b) compared to the control (Fig 8a) was 1.75, just over 1 S.D. from the mean upregulation found by flow cytometry (Table 2), with the greatest increases in fluorescence appearing at the plasma membrane.

Additionally, blocking integrin β_1_ during the healing phase following exposure to low shear provided evidence of the potential role β_1_ may play in conjunction with actin filament reorganization to promote wound healing. The blocking antibody was added to the cell medium after 24 hours of low shear, and healing was compared to a no-shear and shear control (without the antibody). After one day, blocking β_1_ integrin in cells exposed to low shear impaired wound closure: 15% of the wound was healed compared to 59% without β_1_ blocking. Blocking β_1_ in shear-exposed cells led to healing similar to no-shear control cells (16% healed) at day 1, while at day 2, the β_1_-blocked sample had healed more than the control cells (45% versus 25%, respectively), and the non-β_1_ blocked shear sample was fully healed (n = 2). These results highlight the role of integrin β_1_ in wound healing following shear, although further research will be needed to fully elucidate this mechanism.

ICAM-1 is a transmembrane molecule that is related to the cytoskeleton [57], focal adhesion [58] components, as well as the inflammatory response of corneal epithelial cells through leukocyte-epithelial cell interactions [59,60]. It has also been shown that ICAM-1 can be involved in various cell-signaling pathways [61]. In our study, as shown in Table 2, exposure to shear stress downregulated ICAM-1 expression, and expression in cells exposed to 4 dyn/cm^2^ was significantly lower compared to the control (no shear). ICAM-1 has been previously linked to cytoskeletal proteins and components such as α-actinin and actin filaments [57]. Jilkova *et al* [54] and Morigi *et al*. [62] have shown that for endothelial cells, cytoskeleton rearrangement in response to shear stress of up to 37 dyn/cm^2^ was accompanied by an upregulation in ICAM-1 expression. The ICAM-1 downregulation observed in the presence of shear could suggest that the mechanism of ICAM-1 expression in response to shear stress differs between corneal epithelial and endothelial cells. ICAM-1 promotes leukocyte-epithelial cell interactions [59,60] which would be detrimental to the ocular surface in the absence of inflammation. The observed ICAM-1 downregulation upon exposure to shear stress is interesting in the context of ocular changes that may be induced during open and closed-eye (sleep) conditions and warrants further investigation.

### Exposure to external shear stress increases the number of apoptotic and necrotic HCECs

**Fig 9:**
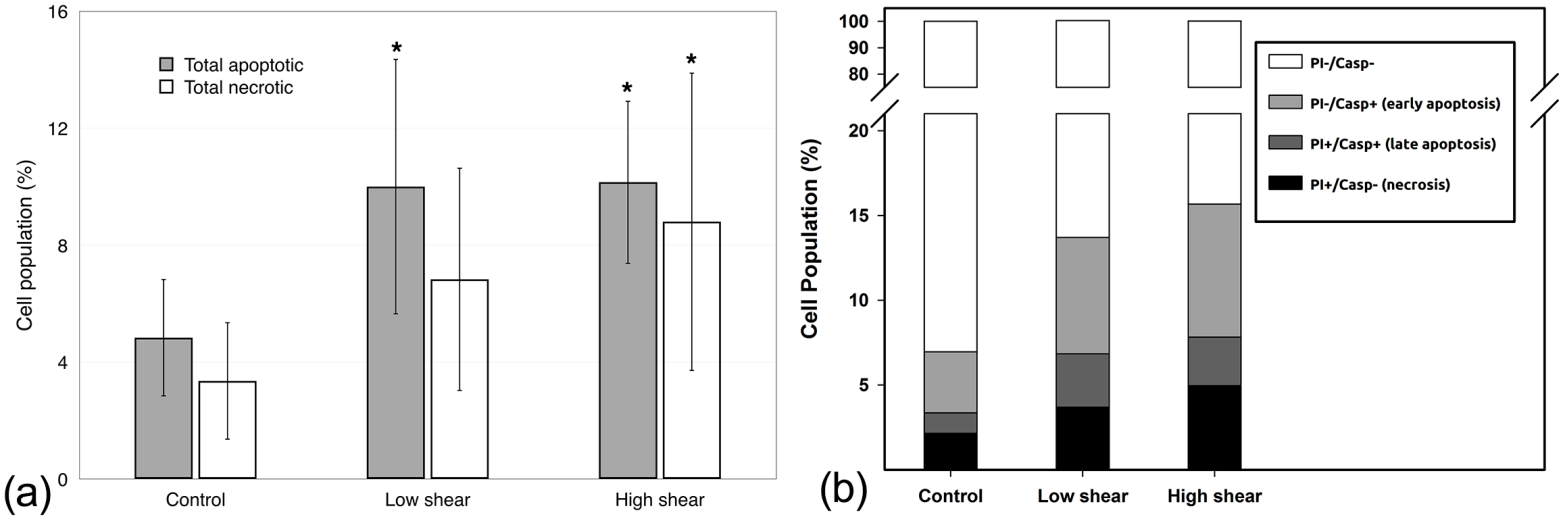
The effect of shear stress on apoptosis and necrosis. a) Total apoptotic and necrotic cells; mean ± SD. b) Distribution of cell population (mean values only; standard deviation has been omitted for clarity). HCECs were exposed to 0 (control), 4 (low) and 8 (high) dyn/cm^2^ shear stress for 24 hours. Caspase-mediated apoptosis was measured with FAM-VAD-FMK and PI to detect necrotic cells. * *p* < *0*.*04* compared to control, n = 5 to 6.

To further characterize the effect of shear stress on HCECs, apoptosis and necrosis were investigated. Exposure of HCECs to flow-induced shear stress significantly increased the total number of apoptotic and necrotic cells (Fig 9a). The increase in necrotic cells appeared to be dependent on the shear stress magnitude, suggesting increased cell damage with higher shear stress (8 dyn/cm^2^). The change in cell population affected by apoptosis, necrosis and secondary necrosis with increased shear stress is further illustrated in Fig 9b.

Apoptosis is recognized as a highly regulated and systematic cell death mechanism used by the body to remove damaged cells [63] and control cell number and proliferation [64], even during the wound healing process [65]. Previous studies have shown that shear stress can affect apoptosis in different cell types such as endothelial cells [66–68], osteoblasts [8], and tumor cells [9]. In the cornea, Yamamoto *et al*. [69] reported that lens wear and eyelid closure during sleep decreased the number of dead corneal epithelial cells, and suggested that shear stresses induced during blinking may increase the number of dead cells and rate of cell shedding. Increased cell shedding in the cornea due to shear stresses had been shown by Ren and Wilson [24] prior to their report. The current studies were performed using monolayers, making it difficult to draw conclusions on the effect of shear on corneal cell shedding; further investigation using stratified cultures will be required to fully assess the relationship between shear stress and cell shedding.

## Conclusions

Using immortalized and primary human corneal epithelial cells, we demonstrated that HCECs are sensitive to flow-induced shear stress and change their behaviour in response to this mechanical signal. Their response is dependent on both the level of shear stress and exposure time. HCECs significantly reorganized their cytoskeleton when exposed to low shear stress (4 dyn/cm^2^), which in turn affected their migratory behavior and their ability to heal a wound *in vitro*. Higher levels of shear stress (8 dyn/cm^2^) impaired wound healing and increased necrosis. Furthermore, exposing cells to shear stress during wound healing seemed to impair healing rate when the cells had not been previously exposed to shear, and therefore lacked cytoskeletal reorganization. Conversely, shear-exposed cells were capable of maintaining their wound healing “advantage” compared to no-shear cells even when exposed to additional shear during wound healing, further highlighting the importance of shear-induced phenotypic change in corneal epithelial cells. These results further emphasize the need to measure the magnitude of shear stress induced by blinking *in vivo* to aid in development of more accurate *in vitro* models.

## Supporting information

S1 Data The raw data used in this study is provided in the supplementary file in excel spreadsheets. (XLSX)

S1 Movie Movie of fluid flow in the chamber. Water was mixed with red food coloring and passed through the *in vitro* system under experimental conditions similar to our cell experiments for 4 dyn/cm^2^. The movie displays conditions when flow direction is changed during the experiment.
(MP4)
